# Communications

**Published:** 1875-06

**Authors:** 


*Communications.*

Our friends must not during the "warm term" just before us neglect the journal in the important particular of writing for its pages. We trust all friends will trim their "gray goose quills" and go to work. Fill our pages. Let your facts seek the light of day, and go forth to bless your medical brethren. Do not hold your light under a measure. P.

DR. T. S. POWELL is on a visit to his native State, in order to deliver the commencement address before the young ladies of Petersburg Female College. The Doctor goes among his old friends and visits the home of his boyhood. We wish him a pleasant time and a safe return to his home. G.

